# Phonology facilitates deeply opaque logographic writing

**DOI:** 10.1371/journal.pone.0312471

**Published:** 2024-10-30

**Authors:** Mio Yokoi, Kouji Takano, Kimihiro Nakamura

**Affiliations:** 1 National Rehabilitation Center for Persons with Disabilities, Tokorozawa, Japan; 2 Japan Society for the Promotion of Science, Tokyo, Japan; Education University of Hong Kong, HONG KONG

## Abstract

Phonological knowledge plays a pivotal role in many aspects of language processing, but it remains controversial whether it is required for writing. In the present study, we examined the issue by focusing on written production in an opaque logographic script (kanji) with highly irregular pronunciation rules, which allowed for a rigorous test of whether or not phonology contributes to writing. Using a phonological priming paradigm in two experiments, we measured response latency while participants orally named target pictures or wrote down their names in kanji. Each target was preceded by a phonographic character (kana) which represented the same sound (mora) as the beginning of the target name or a different mora. By manipulating the degree of phonological overlap between primes and target names (i.e., morae, consonants and vowels), we found that only the moraic overlap could speed up word production in logographic writing (Experiment 1). In contrast, naming response was facilitated by mora-overlap as well as vowel-overlap. This between-task difference in phonological encoding suggests that phonological codes for spoken production do not necessarily precede orthographic access during logographic writing. In Experiment 2, we further found that the facilitatory effects of moraic information did not differ in magnitude between writing and naming when primes were masked and presented more briefly, suggesting a net component of bottom-up phonological activation which contributes to logographic writing. Collectively, we propose that orthographic codes of kanji are accessed directly from semantics, whereas phonology plays a non-specific modulatory role to enhance neurocognitive systems involved in writing.

## Introduction

Handwriting is a fundamental building block of literacy which can facilitate visual word recognition across different languages [1, 2]. Yet the skill for written production is known to develop much more slowly compared to speech production. While children have the basic phonetic inventory for speech by the age of 5 years [3], it is only through a few years of elementary school education that they acquire an adult-like motor processing skill for spelling (i.e., at 9–10 years of age [4]). As opposed to the extensive research dedicated to speech production, however, relatively less work has been devoted to understanding cognitive mechanisms of writing over the last few decades. It therefore remains poorly understood how spelling retrieval and motor production, that is, the two core processes involved in writing [4, 5], are orchestrated during written production in literate adults. In particular, it is a major unresolved issue whether phonological representations are necessary for written word production. That is, while most cognitive models of writing count the semantic and orthographic memory systems as mandatory components for written production, it remains controversial whether or not phonological memory contributes to the act of writing.

**Fig 1:**
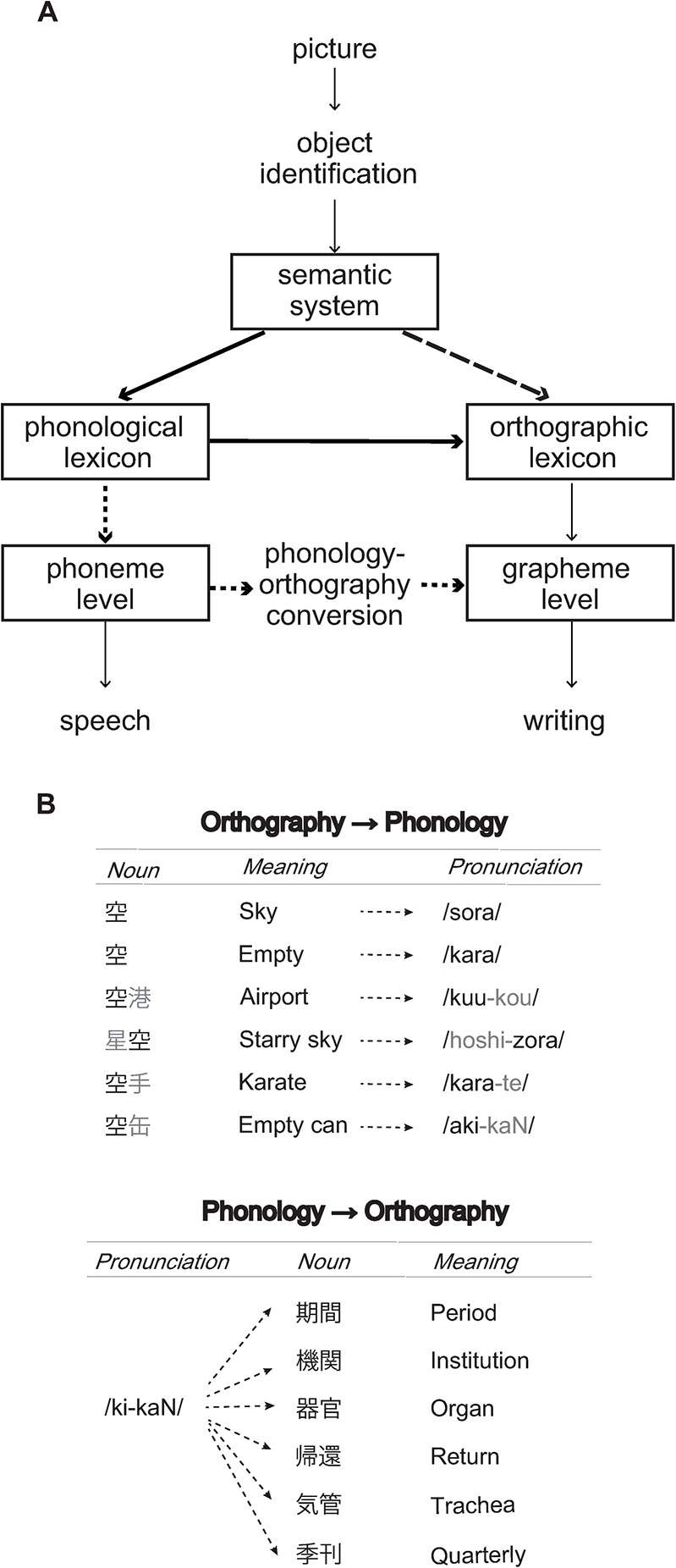
A. Cognitive model of spoken and written naming (modified from Bonin et al. (2011). Spoken and written production systems consist of shared components for visual structural analysis and semantic analysis as well as modality-specific components for generating response outputs, that is, phonological lexicons/ lexemes for naming and orthographic lexicons/lexemes for writing. After common visual structural and semantic analyses, orthographic lexicons/lexemes required for writing may be accessed directly from the semantic system during written production (“orthographic autonomy hypothesis”, dashed line). Alternatively, such orthographic codes may be accessed either via the direct lexical-level link between phonological and orthographic lexicons (solid line) or via the sublexical-level link between phonological and orthographic lexemes (dotted line) (“phonological mediation hypothesis”). B. Orthography and phonology in kanji. The kanji script is a highly opaque writing system whose correspondence between form and sound is one-to-many in both directions. That is, each character (e.g., “空”) can represent, either on its own or in conjunction with other characters, several different sounds and meanings (top). On the other hand, the same sound (e.g., “kikaN”) can be mapped onto several different words and meanings, each written with different kanji characters (bottom).

More specifically, there are two opposing views about whether phonology is mandatory for accessing orthographic codes required for writing (Fig 1A). Early neuropsychological models assumed that orthography can be accessed only via the prior retrieval of phonological representations [6, 7]. According to this view, orthographic outputs are generated by translating lexical or sublexical phonological codes activated from the semantic system [7, 8]. This “phonological mediation hypothesis” seems to be compatible with the fact that various spelling errors occur in association with phonology in both alphabetic and logographic scripts. For example, English words like "consensus" and “recommend” are often misspelled as “concensus” and “recomend”, respectively. Similar phonological errors are also known to occur in logographic writing, for example, the word社会 /sha.kai/ (company) in Japanese is occasionally misspelled as 社回/sha.kai/ (nonsense combination) [9]. In fact, these observations have been substantiated by more empirical studies in alphabetic [10] and logographic [11, 12] languages. Namely, Bonin et al. (2001) used a graphics tablet to measure written latency while French participants performed a written naming task and showed that written responses were faster when target names were consistent in sound-to-spelling mapping than when otherwise. Using a picture-word interference task with Chinese participants, Qu et al. (2015) also showed that written word production is faster when distractors shared initial syllables with target names than when otherwise. These results thus suggest that phonological codes play a mandatory role in generating written outputs irrespective of the nature of writing systems.

An alternative view, or “orthographic autonomy hypothesis”, has been put forward by neuropsychological studies of writing disorders [13, 14]. For instance, Rapp et al. (1997) described an individual who was able to produce written, but not spoken, names of visual objects in English. A similar dissociation between speaking and writing was also reported for logographic Chinese [14]. These observations are taken as suggesting that orthographic lexical forms can be accessed without the mediation of phonology during written production. This orthography autonomy hypothesis has gained further support from more recent behavioural studies with healthy participants in both alphabetic [15] and logographic [16] writing systems. Taken together, these results suggest that orthographic word forms are autonomous from phonological forms and can be activated directly from semantic information.

Given the current status of the two competing hypotheses as described above, it remains largely open whether phonology contributes to writing. In the present study, we conducted two primed naming experiments to examine response latency while Japanese participants made written naming in logograms (kanji). It is important to note that Japanese kanji is a highly opaque writing system, of which the mapping between print and sound is one-to-many in both directions, whether from print to sound or vice versa (Fig 1B) and is thus much more irregular than the one in Chinese, where each character has typically only one way of reading. It seems therefore plausible that orthographic codes of kanji words need to be accessed directly from semantics and without the mediation of phonological information during writing. Nevertheless, it is also possible that the opaque logographic system is strongly associated with sound—for example, a masked priming study by Verdonschot et al. (2013) has shown that reading of a kanji automatically activates multiple pronunciations in Japanese participants [17]. Therefore, if phonological information exerts a measurable impact on such opaque logographic writing, it may provide additional support for the contribution of phonology in writing.

**Fig 2:**
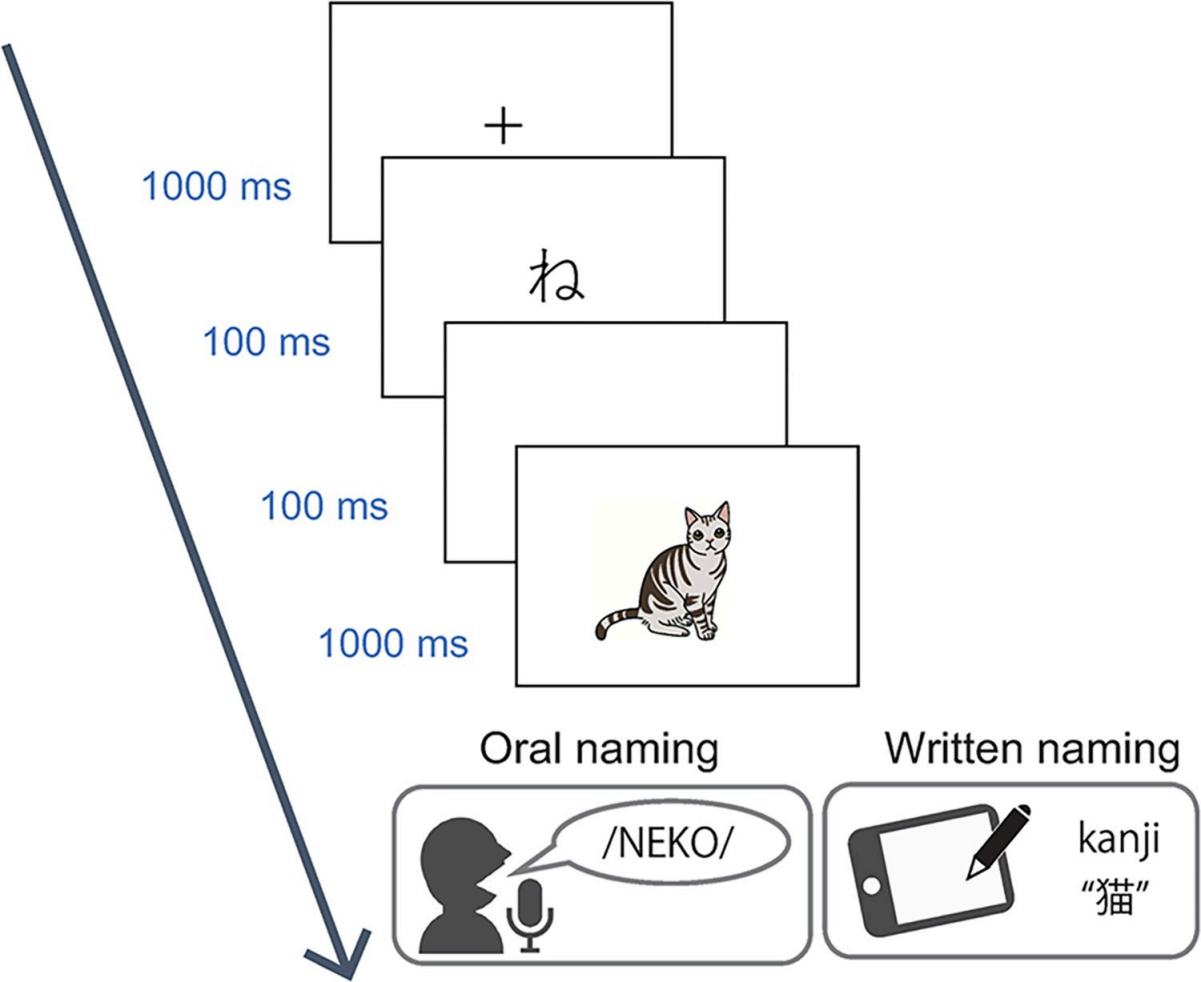
Behavioral paradigm. Each trial consisted of a fixation cross, a prime (single kana character), a blank interval and a target picture. Participants either orally named the targets or wrote down their names according to prespecified task instructions. Four different types of primes were used to manipulate the degree of phonological overlap between primes and the first morae of target names. That is, each prime could represent either (1) a mora identical to the first mora, or (2) a different mora sharing only a consonant with the first mora, or (3) a different mora sharing only a vowel with the first mora, or (4) a different mora sharing neither consonants nor vowels with the first mora (see Methods).

Specifically, we used a phonological priming paradigm to measure response latency during written naming (Fig 2). On each trial, participants viewed target pictures preceded by kana (moraic) character primes and then either orally named the targets or wrote down their names. Here, the kana script is a highly regular writing system with 46 basic characters, each of which has almost one-to-one correspondence with sound (see Methods for detail) and thus serves as visual primes carrying critical phonological information. By manipulating the degree of phonological overlap between primes and target names (i.e., morae, consonants, vowels and different morae), we examined whether phonological information can influence written naming in kanji (Experiment 1). We used oral naming as a control task to measure the magnitude of phonological activation during word production, because the phonological overlap at initial morae is shown to speed up spoken production in Japanese by partially activating phonological codes during the early stages of phonological encoding (e.g., [18, 19]. The four different types of primes were used because it seems unknown whether the functional unit of word production is identical between written and spoken modalities (see Methods). Comparing the effects of phonological priming between the two response modalities therefore allowed for a stringent test of whether or not phonology contributes to writing see [16, 20, 21]. Specifically, if such phonological codes mediate the generation of orthographic outputs at the lexical or sublexical level (see Fig 1A), the prime-target phonological overlap will facilitate response production not only in naming but also in writing. Conversely, if phonology does not contribute to the access of orthographic codes during writing, those lexical or sublexical phonological codes are functionally irrelevant to the act of writing, such that facilitatory effects of phonological priming should be found for naming but not for writing. By comparing the effects of phonological overlap between the two tasks, we examined possible differences in phonological encoding between written and spoken production, since the functional phonological unit in word production may change with the nature of stimuli and tasks e.g., [22, 23]. In Experiment 2, we further used a standard “masked priming” paradigm to examine whether or not phonological information affects written production when top-down, strategic processing of primes is minimized by visual masking.

## Experiment 1

### Method

#### Participants

Thirty-one native Japanese speakers (10 males, age 18–45 years, mean age 30.06 years) were recruited between 5 December 2022 and 30 June 2023. All had normal or corrected-to-normal vision and received at least 12 years of formal education. The protocol of the present study was approved by the ethical review committee of the National Rehabilitation Center for Persons with Disabilities (#2021–135). All participants provided written informed consent prior to the experiments. All experiments were performed in accordance with the Declaration of Helsinki.

#### Materials and procedures

Seventy-four object pictures (animals, commodities, foods, fruits, plants, vehicles etc.) were used as targets for the writing and naming tasks (see Fig 2). Since the existing normative database for Japanese e.g., [24] included only a limited number of pictures whose names are normally written in kanji, we collected easily identifiable colored object drawings from freely available resources on the internet (see https://osf.io/yhe9x/ for stimulus materials). All object names consisted of 2–4 morae (mean = 2.9). Thirty-five of them were written with one character in kanji, while the remaining with two kanji characters.

Four different types of prime-target pairs were created for each of the 74 object pictures. First, “mora-overlap” pairs were generated by coupling each target with a single kana character which represented the first mora of the target names (e.g., prime = ね /ne/, target = ねこ /neko/ (“cat”)). In general, kana characters each represent a “mora”, or a syllable-like unit of phonological timing used for speech production in Japanese [18, 19, 25]. Thus, the vowels used as mora-overlap primes each were identical to the initial vowels of target words. Second, we created “consonant-overlap” pairs in which primes shared the initial consonants with target names (e.g., prime = そ /so/, target = さかな /sakana/ (“fish”)). Third, we also created “vowel-overlap” pairs in which primes shared the initial vowels with targets (e.g., prime = は /ha/, target = まど /mado/ (“window”)). Forth, we created “different mora” pairs in which primes shared neither consonants nor vowels with the first mora of target names (e.g., prime = ね /ne/, target = くも /kumo/ (“cloud”)). The four different types of prime-target pairs were used to manipulate the degree of phonological overlap between primes and targets, because it is largely unknown whether the functional unit of word production is identical between written and spoken modalities. That is, while the functional unit of speech production is known to vary among different languages (e.g., a phoneme for English and Dutch, a syllable for Chinese [26] and a mora for Japanese [27], it seems open to what extent the same phonological units contribute to written production in each language.

The 74 prime-target pairs were assigned to four sets of 18–19 items (i.e., 2 sets with 19 items and 2 sets with 18 items) matched in numbers of characters, morae and strokes, lexical frequency, phonological imageability and orthographic plausibility (*ps* > 0.13 for all) according to the NTT database [28]. We then constructed four presentation lists in which the four sets of prime-target pairs were rotated such that any possible confounding factors are counterbalanced across the four presentation lists. Participants were assigned one of the four lists with which they performed the same two tasks. The experiment consisted of the writing and naming tasks each of which included two blocks of 74 trials (148 trials in total).

Stimulus presentation and data collection were achieved using PsychoPy 3.2.4 [29]. As shown in Fig 2, each trial consisted of central fixation cross (1000 ms), a prime (100 ms) and blank (100 ms) followed by a target (1000 ms). Participants were told that they would see target pictures preceded by single kana characters on each trial. They named target pictures as quickly as possible in the naming task. Oral responses were recorded with a headset microphone. Likewise, participants wrote down target names in kanji as quickly and as accurately as possible in the writing task. Writing responses were recorded using a graphics tablet (Intuos Pro, Wacom, Japan). Participants could not see written outputs throughout the experiment. The effect of task order was counterbalanced across participants. To ensure that participants know correct target names, they performed a training session prior to the main experiment, in which they orally named through all the 74 items and received feedback for correct and incorrect responses.

It is important to note that participants could clearly see prime stimuli because the duration of the primes, as well as that of the subsequent blank period, is much longer than the one used for typical masked priming experiments (30–60 ms). We deliberately used those clearly visible primes in the present experiment so that phonological codes could be fully activated prior to word production. This is important to determine whether phonological information influences the act of logographic writing, because masked primes with shorter duration are likely to activate the phonological system only to a limited degree [30, 31]. In fact, some masked priming studies show that crossmodal phonological activation by masked words can emerge only when the duration of primes exceeds ~65 ms [32–34], see Experiment 2 for further discussion.

### Results

Reaction time (RT) data for incorrect responses or shorter than 200 ms were excluded from analysis. Raw RT data were then log-transformed to reduce the variances for each task and compare the effects of phonological overlap between different tasks. We used the “different mora” condition as the baseline, because such unrelated primes can equate the levels of underlying cognitive processing load across different types of primes and thus are used commonly in priming studies of visual word processing (see e.g., [35]). For the writing task, responses latencies below or above 2 SD from the grand mean (4.5%) were excluded, leaving a total of 4255 data points for analysis. For the naming task, log-RT outliers (4.8%) were also excluded, leaving a total of 4233 data points for analysis. Error rates and RT data for each task are presented as a function of phonological overlap in Table 1.

**Table 1:** Mean response latencies and error rates as a function of phonological overlap for each task in Experiment 1.

Naming				
Prime Type	Mora	Consonant	Vowel	Different
RT (ER)	773 (1.1%)	833 (2.0%)	827 (2.5%)	838 (1.9%)
PE (vs. Different)	65 (0.8%)	5 (-0.1%)	11(-0.6%)	-
Writing				
Prime Type	Mora	Consonant	Vowel	Different
RT (ER)	830 (1.3%)	874 (3.0%)	874(3.1%)	874 (3.0%)
PE (vs. Different)	44 (1.7%)	0 (0%)	0 (-0.1%)	-

Note. RT, ER and PE stand for mean response latencies, error rates and priming effects, respectively. RTs are presented in milliseconds.

Log-transformed RT data were fitted with liner mixed-effects (LMEs) models using the lme4 package [36] implemented in the R environment [37]. We started with the maximal random-effects structure [38], which included fixed effects of task (naming and writing), phonological overlap (“morae”, “consonants”, “vowels” and “different”), task-order (writing-naming and naming-writing) and their interaction as well as by-participant and by-item random intercepts and slopes for all the fixed effects. The three explanatory variables were centered using effect coding to calculate coefficient estimates for testing the main effects and their interaction. By removing random slope terms in a stepwise manner, we simplified the initial model to determine the maximal model that converged for the present dataset. The final model included fixed effects of task, phonological overlap, task-order and their interactions as well as by-participant random intercepts and slopes for the effects of task and task-order. The significance of the main effects and their interactions was assessed with a type III ANOVA via Satterthwaite’s approximation in the lmerTest package [39]. We found that the effects of task and phonological overlap were both significant (*F* (1, 28) = 5.62, *p* = 0.025 and *F* (3, 8345) = 140.05, *p* < 0.001, respectively) and interacted with other (*F* (3, 8342) = 3.98, *p* = 0.008). The effect of task-order was non-significant (*F* (1, 28) = 1.39, *p* = 0.247) and did not interact with other factors (*p* > 0.5 for all)

For each task, we then performed pairwise comparisons between the four levels of overlap (i.e., mora, consonant, vowel and different). All pairwise tests were corrected for false discovery rate (FDR) using the *emmeans* package (https://cran.r-project.org/web/packages/emmeans). Pairwise comparisons for the writing task revealed that participants responded faster in mora-overlap than in other types of trials (*b* = 0.053, *t* = 6.20, *p* < 0.001, vs. consonants; *b* = 0.052, *t* = 6.18, *p* < 0.001, vs. vowels; *b* = 0.053, *t* = 6.26, *p* < 0.001 vs. different morae). Other pairwise comparisons for writing latency were non-significant (*ps* > 0.98 for all). On the other hand, pairwise comparisons for the naming task also showed that participants responded faster in mora-overlap trials than in other types of trials (*b* = 0.074, *t* = 12.09, *p* < 0.001, vs. consonants; *b* = 0.067, *t* = 10.99, *p* < 0.001, vs. vowels; *b* = 0.081, *t* = 13.31, *p* < 0.001, vs. different morae). Participants respond faster in vowel-overlap trials than in different mora trials (*b* = 0.014, *t* = 2.28, *p* = 0.034). Naming latency differed neither between consonant-overlap trials and different-mora trials nor between consonant-overlap trials and vowel-overlap trials (*ps* > 0.27 for both).

We selected those contrasts which survived pairwise comparisons in each task and compared the effects of phonological overlap between writing and naming by testing the interaction with the effect of task (FDR-corrected for multiple comparisons). Between-tasks comparisons showed that the facilitatory effects of morae (relative to the different) was greater for naming than for writing (*b* = 0.028, *t* = 2.70, *p* = 0.028, vs. different morae). No other between-tasks comparisons were significant (*ps* > 0.08 for all).

### Discussion

Results from the writing and naming tasks showed the effects of phonological priming by moraic overlap (i.e., participants responded faster in mora-overlap trials relative to other trials). This finding thus seems consistent with the notion that the basic functional unit of phonological encoding is the mora in Japanese [27]. More interestingly, we found that the facilitatory effect of vowel overlap was significant for naming. This finding may be unexpected, not only because the functional unit of speech production is thought to be the mora in Japanese [27] but also because such facilitatory effects of vowels on spoken production seem to have been rarely reported in other languages as well. However, a few relevant observations can be found in the literature. For example, a picture-word interference study by Lupker (1982) showed that naming response was facilitated when target pictures (e.g., “plane”) were presented with clearly visible words having shared vowels (e.g., “brain”) with target names than when presented with unrelated words (e.g., “power”) [40]. Meyer and Schriefers (1991) also observed a similar effect of phonological facilitation by shared vowels in a crossmodal picture-word interference paradigm [41]. Such response facilitation, termed as “phonological similarity effect”, may be responsible for the observed effects of vowel-overlap on naming in our experiment. In fact, the phonological similarity of words is also known to impact word processing in Japanese. For example, Saito et al. (2008) showed that a pair of words are more often confused with each other when they are phonologically similar (e.g., 次 (“tugi”) and 茎 (“kuki”)) than when otherwise (e.g., 春 (“haru”) and 丘 (“oka”)) [42].

We used clearly visible primes in the present study so that the phonological system should be fully activated because we wanted to determine whether phonological information influences the act of logographic writing. However, given the reliable effects of phonological priming in Experiment 1, it is interesting to ask whether phonology also affects written production in a standard masked priming paradigm where top-down, strategic influences are minimized by visual masking. As argued above, phonological processing as well is likely to be restricted in such experimental settings, since crossmodal phonological activation by masked words seems to occur when the duration of primes exceeds 65 ms in visual word recognition [32, 33] as well as in naming [34]. According to these observations, it should be expected that oral naming will be facilitated when masked primes with phonological overlap are presented for 65 ms or more. We examined whether this is also the case for logographic writing in Experiment 2.

## Experiment 2

### Method

#### Participants

Twenty-seven native Japanese speakers (13 males, age 18–50 years, mean age 27.92 years) were recruited between 5 December 2022 and 25 August 2024. None of them participated in Experiment 1.

#### Materials and procedures

All materials and procedures were identical to Experiment 1, except that (1) primes were presented for 67 ms and (2) they were preceded by a forward mask (##) for 500 ms and followed by a backward mask (&&) for 67 ms. Target pictures were presented immediately after the backward mask.

#### Results and discussion

For the writing task, incorrect responses and responses latencies below or above 2 SD from the grand mean (6.2%) were excluded, leaving a total of 3745 data points for analysis. For the naming task, incorrect responses and outliers (i.e., log-RT > 2 SD, 6.1%) were also excluded, leaving a total of 3715 data points for analysis. Error rates and RT data for each task are presented as a function of phonological overlap in Table 2. We started with the same initial model as in Experiment 1 and found the final model that included fixed effects of task, phonological overlap, task-order and their interactions as well as by-participant and by-item random intercepts and slopes for the fixed effect of task. The effects of task and phonological overlap were both significant (*F* (1, 29) = 19.02, *p* < 0.001 and *F* (3, 7252) = 17.68, *p* < 0.001, respectively). The effect of task-order was neither significant (*F* (1, 25) = 0.85, *p* = 0.366) nor interacted with other factors (p > 0.35 for all). Unlike in Experiment 1, the interaction between task and phonological overlap was non-significant (*F* (3, 7257) = 0.07, *p* = 0.977), suggesting that overall effects of phonological overlap did not differ between writing and naming.

**Table 2:** Mean response latencies and error rates as a function of phonological overlap for each task in Experiment 2.

Naming				
Prime Type	Mora	Consonant	Vowel	Different
RT (ER)	751 (1.7%)	770 (1.6%)	773 (2.0%)	768 (2.0%)
PE (vs. Different)	17 (0.3%)	-2 (0.4%)	-5 (0.0%)	-
Writing				
Prime Type	Mora	Consonant	Vowel	Different
RT (ER)	846 (2.0%)	863 (2.2%)	870 (3.2%)	862 (2.2%)
PE (vs. Different)	16 (0.2%)	-1 (0.0%)	-8 (-1.0%)	-

Accordingly, we performed pairwise comparisons between the four levels of overlap by entering all RT data from the two tasks. The facilitatory effects of mora-overlap were again significant relative to other types of trials (*b* = 0.022, *t* = 3.66, *p* < 0.001, vs. consonants; *b* = -0.029, *t* = -4.71, *p* < 0.001, vs. vowels; *b* = 0.020, *t* = 3.27, *p* = 0.001, vs. different morae). Other pairwise comparisons were non-significant (*ps* > 0.15 for all).

Collectively, the present results show that the moraic overlap between primes and targets can facilitate both naming and writing even when strategic or controlled cognitive components are restricted by visual masking. The observed effects of mora-overlap during naming are consistent with the masked priming study by Chetail and Mathey [34] and likely to arise at the early stage during word retrieval because the effects of masked primes are thought to dissipate in 200 ms from stimulus onset [43, 44]. Our results further suggest that such fast and transient neural activation by masked primes can also help written production although the act of writing is more time-consuming and strategic in nature as compared to spoken production (e.g., [16, 20, 45]).

Accordingly, the present results suggest that there is a net component of bottom-up phonological activation which contributes to logographic writing. Interestingly, the observed lack of task x phonological overlap interaction suggests that such bottom-up activation of phonological codes does not differ in magnitude between writing and naming. In turn, strong effects of mora-overlap observed in Experiment 1can be attributed in large part to late-stage neurocognitive components involved in writing, because the magnitude of phonological priming was substantially reduced in Experiment 2 (15 ms) as compared to Experiment 1 (55 ms). Indeed, clearly visible primes with longer duration as used in Experiment 1 are thought to produce strong and durable activation in multiple brain areas [30, 31] (see also General Discussion).

## General discussion

Results from Experiment 1 revealed that the phonological congruency between primes and targets affects response latency not only in naming but also in writing in kanji, whereas this effect was the most pronounced when primes and targets shared initial morae. This finding therefore suggests that mora-level phonological information exerts a significant impact on written production even in the highly opaque logographic script. Our results overall concur with the previous study showing that masked primes sharing initial syllables with target names facilitate written word production in logographic Chinese [12]. Since character-to-sound correspondence is much more irregular in Japanese kanji, the present results extend the previous research by showing that phonology plays a role in handwriting even in the more deeply opaque logographic script. This interpretation also seems compatible with the fact that many spelling errors in kanji occur in association with phonology, rather than with lexico-semantics, in normal adults, typically developing children and children with dyslexia [9, 46, 47].

Accordingly, the present results are seemingly at odds with the orthographic autonomy hypothesis and more consistent with the phonological mediation hypothesis according to which orthographic access relies on the prior retrieval of phonological representations. This interpretation, however, does not fit well with the fact that print-to-sound mapping in kanji is one-to-many in both directions (see Fig 1B), such that phonological activation induced by primes is unlikely to help when accessing orthographic codes. In fact, while we observed in Experiment 2 that the magnitude of masked phonological priming by overlapping morae does not differ between writing and naming, this finding does not necessarily support the notion that phonology precedes orthographic access during writing. Rather, as argued below, bottom-up activation of phonological codes may act at other stages of written production. Collectively, neither of the two competing hypotheses seems to fit well with the findings from the present experiments.

Conversely, if phonological activation does not serve orthographic access, how can we account for the observed facilitatory effects of mora priming during kanji writing? A possible account is that (1) orthography can be directly accessed from semantics but (2) this semantic-orthography route is not fully autonomous from phonology such that it can be enhanced by additional phonological information during written production. Empirical support for the latter point is provided by neuroimaging data showing that conscious phonological coding of written words can drastically increase the activation level of the cerebral reading network ([48], see also [30, 31] for review). Since reading and writing rely on shared neural systems in the left hemisphere [49, 50], additional phonological activation by congruent primes may elevate the global activation level of these neural systems and their functional connection strength during writing, thereby facilitating written production at multiple stages from semantic analysis via orthographic access down to final motor execution. Such global amplificatory influences may be analogous in nature to “contextual” effects induced by various task-relevant cues which can facilitate word production (e.g., [51–53]). Our results thus can be taken as suggesting that phonology plays such modulatory and auxiliary role in written production in kanji, rather than mediating orthographic access per se.

Notably, a relevant neurocognitive mechanism may be found in the “graphemic buffer system”, i.e., a working memory component of the spelling system which seems to play a universal role in written production across alphabetic [54–58], syllabic [59] and logographic [60, 61] writing systems. Such graphemic buffer is thought to mediate written word production by temporally holding an ordered sequence of abstract character identifies during the generation of motor sequences for writing and rely on a broad left-hemisphere network, including the frontoparietal, lateral occipital and subcortical regions [62]. Thus, although print-to-sound mapping is highly irregular in kanji, overlapping morae may broadly pre-activate these neural buffering mechanisms as compared to different morae, thereby facilitating the subsequent retrieval of hand motor sequences required for written production.

In addition, there are a few other findings worthy of note in the present study. Namely, we observed that naming was faster in vowel-overlap trials relative to different mora trials in Experiment 1. This facilitatory effect of vowels on naming might be worthy of further investigation since there seem to be almost no previous studies that reported similar observations in the masked priming literature. While such positive effect of vowel overlap was never found for writing across the two experiments, this should be also the case for naming if the functional phonological unit for word production is the mora in Japanese, rather than the phoneme in other languages like English and Dutch [25]. However, it is possible that other phonological units, rather than the mora, contribute to speech production in Japanese depending on the nature of stimuli and tasks. For example, Yoshihara et al. (2017) showed that the phonological unit used for naming kanji compounds is coarser than the mora, that is, the whole sound of each kanji character [22]. On the other hand, Kureta et al. (2015) showed that phonological coding in spoken production can occur at a finer, phonemic level when Japanese words are spelled in Latin script (“romaji”) such that participants are aware of the initial phonemes of the stimuli [23]. The functional units of phonological coding may also change with task demands during word production, because we also observed that patterns of phonological priming differed between writing and naming in Experiment 1. Moreover, phonological priming may occur simultaneously at different levels, because for instance, Chen et al. (2016) showed that prime-target overlap at initial phonemes produced a negative impact on naming latency in Chinese, where the syllable, rather than the phoneme, is thought to act as the functional unit of speech production [26]. Therefore, although the question itself is beyond the scope of the present study, further research is needed to investigate the possible change in the functional units of speech production in each language.

To summarize, the present results show that phonological codes contribute to written production even in the deeply opaque kanji script where each character may have different ways of reading. This is seemingly inconsistent with the view that orthographic codes can be accessed without the mediation of phonology during writing. However, our results also suggest that phonology does not necessarily precede orthographic access during writing in kanji. Although the present design does not allow us to isolate the specific cognitive locus generating the facilitatory effects of phonology on writing, our results are more compatible with another possibility that orthographic codes of kanji are accessed directly from semantics, where phonology plays a non-specific modulatory role to enhance neurocognitive systems involved in writing. Obviously, this tentative account needs to be examined more systematically in future research, possibly by the joint use of more rigorous behavioral testing with functional brain imaging.
